# Fluoroscopy-guided trans-urethral exchange of double-J ureteral stents

**DOI:** 10.1186/s12894-022-01034-3

**Published:** 2022-06-15

**Authors:** JungWon Kwak, Sung Bum Cho

**Affiliations:** Department of Radiology, Korea University Anam Hospital, 73, Goryeodae-ro, Seongbuk-gu, Seoul, Korea

**Keywords:** Ureteral stent, Fluoroscopy, Malignant ureteral obstruction

## Abstract

**Background:**

For patients with malignant ureteral obstruction or stricture who require long-term internal drainage, plastic double-J stents (DJ stents) represent the mainstay of therapeutic strategies. DJ stents should be replaced at least once every 6 months to avoid infection or obstruction. Although DJ stents are generally replaced under cystoscopy, successful fluoroscopy-guided retrograde replacement of DJ stents in the interventional suite has been described in the literature.

**Methods:**

Between April 2004 and May 2020, we exchanged 143 DJ stents in 19 male and 22 female patients under fluoroscopic guidance using Nelaton catheters, snare catheters, and 8F DJ stents. All procedures were performed with patients under sedation and local anesthesia. There were 39 patients with malignant ureteral obstruction and two patients with benign ureteral strictures. This study was approved by the Institutional Review Board. Technical success, clinical success, complications, procedure time, and mean interval between two procedures were retrospectively reviewed, and the factors affecting the success rate of the procedure were analyzed.

**Results:**

Obstruction was detected at the abdominal ureter in 4 patients, pelvic ureter in 29 patients, and intravesical ureter in 8 patients. Twenty-six patients underwent two or more sessions of the procedures, whereas 15 patients underwent single-session procedures. Total 34 outpatient-based procedures and 109 inpatient-based procedures were performed. Technical success and clinical success were achieved in 94.4% (135/143) and 93.3% (126/135) procedures, respectively. Mean procedure time was 21.5 min (range 9–192 min). Mean procedure interval was 101.8 days (range 5–306 days). Technical success was negatively affected by male sex and obstruction at the pelvic ureter and was positively affected by previous successful exchange. Left-sided ureteral stent placement and old age negatively influenced clinical success. Septic shock occurred in one patient and was treated with antibiotics.

**Conclusion:**

Fluoroscopy-guided trans-urethral exchange of DJ stents is an effective and less painful procedure.

## Introduction

Generally, percutaneous nephrostomy and ureteral stent insertion are performed to achieve urinary tract decompression in patients with urinary tract obstruction. For patients with malignant ureteral obstruction or stricture who require long-term internal drainage, plastic double-J (DJ) stents represent the mainstay of therapeutic strategies [1, 2].

DJ stents should be replaced at least once every 6 months to avoid infection or obstruction [1, 2]. Although DJ stents are generally replaced under cystoscopy, successful fluoroscopy-guided retrograde replacement of DJ stents in the interventional suite has been described in the literature [1, 2].

The aim this study was to evaluate the outcomes of fluoroscopy-guided trans-urethral exchange of DJ ureteral stents.

## Materials and methods

### Patients

Between April 2004 and May 2020, the authors exchanged 143 DJ stents in 41 patients (19 men and 22 women), with a mean age of 63.9 years (range 33–84 years). There were 16 patients with gynecologic cancers, 10 patients with urogenital cancers, 10 patients with gastrointestinal tract cancers, one patient with breast cancer, one patient with pelvic fibrosarcoma, one patient with retroperitoneal liposarcoma, and two patients with benign ureteral strictures (Table 1).

**Table 1:** Patient characteristics

Characteristics			
	N	Mean	Range
Total patients	41		
Male	19		
Female	22		
Age, years		63.9	33–84
Total stents	143		
Unilateral stents	65		
Bilateral stents	78		
*Number of procedure*			
1	15		
≥ 2	26	3.3	2–7
Interval between procedures, days		101.8	5–306
*Underlying conditions*			
Gynecological cancer	16		
Urogenital cancer	10		
GI tract cancer	10		
Breast cancer	1		
Pelvic fibrosarcoma	1		
Retroperitoneal liposarcoma	1		
Benign strictures	2		
*Obstruction level*			
Abdominal ureter	4		
Pelvic ureter	29		
Intravesical ureter	8		

Initially, three patients underwent retrograde implantation of DJ stents under cystoscopy, whereas 38 patients underwent anterograde implantation of DJ stents via a percutaneous nephrostomy route. Among these 38 patients, seven were referred by urologists because of initial retrograde implantation failure. This study was approved by the Institutional Review Board (IRB) (approval number: 2021AN0230).

### Technique

All procedures were performed with patients under sedation and analgesia. Intravenous antibiotic prophylaxis was administered immediately before the procedure. All procedures were performed in the angiography suite (Artis Q; Siemens Healthcare, Forchheim, Germany) under fluoroscopic guidance. Patients were placed in the supine position. Patients’ urogenital region was sterilized with Betadine.

**Fig. 1:**
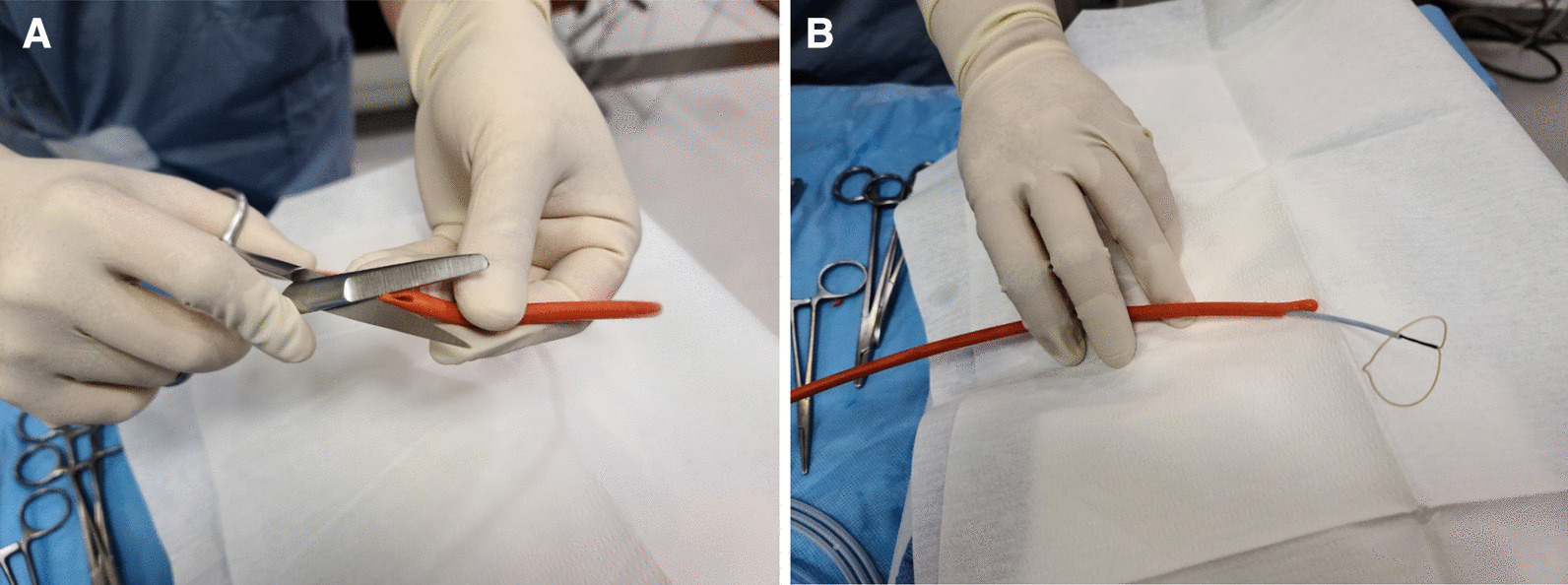
**A**, **B**. Photographs showing the use of scissors to expand the side hole at the distal tip of an 8F Nelaton catheter (**A**) to allow the passage of a 5F snare catheter through the side hole (**B**)

After widening the side hole at the distal tip of an 8F Nelaton catheter (Sewoon Medical, Cheonan, Korea) to allow the passage of a 5F snare catheter (Gooseneck snare; ev3 Inc., Plymouth, MN, USA) (Fig. 1), lidocaine gel was applied topically to patients’ urethra through the Nelaton catheter under local anesthesia. The Nelaton catheter was inserted into the bladder through the urethra.

Then, the bladder was filled with diluted intravenous contrast material (ratio of contrast material to saline 1:9). The endovascular snare catheter was inserted into the bladder via the Nelaton catheter. The distal J of the DJ ureteral stent was grabbed and withdrawn to the urethral orifice. Then, the distal ureteric stent was cannulated with a hydrophilic 0.035-inch guidewire (Terumo, Tokyo, Japan), which was introduced through the stent to the renal pelvis. The ureteric stent was removed over the guidewire, and a 5Fr Kumpe catheter (JS Medical, Seoul, Korea) was advanced over the guidewire into the renal pelvis. Pyelograms were obtained after injecting contrast material into the renal pelvis through the Kumpe catheter to evaluate renal pelvis status (e.g., the presence of stone and debris). The stiff 0.035-inch guidewire was exchanged with a soft 0.035-inch guidewire (Terumo, Tokyo, Japan). Next, the Kumpe catheter was withdrawn while the stiff guidewire was left in the renal pelvis.

**Fig. 2:**
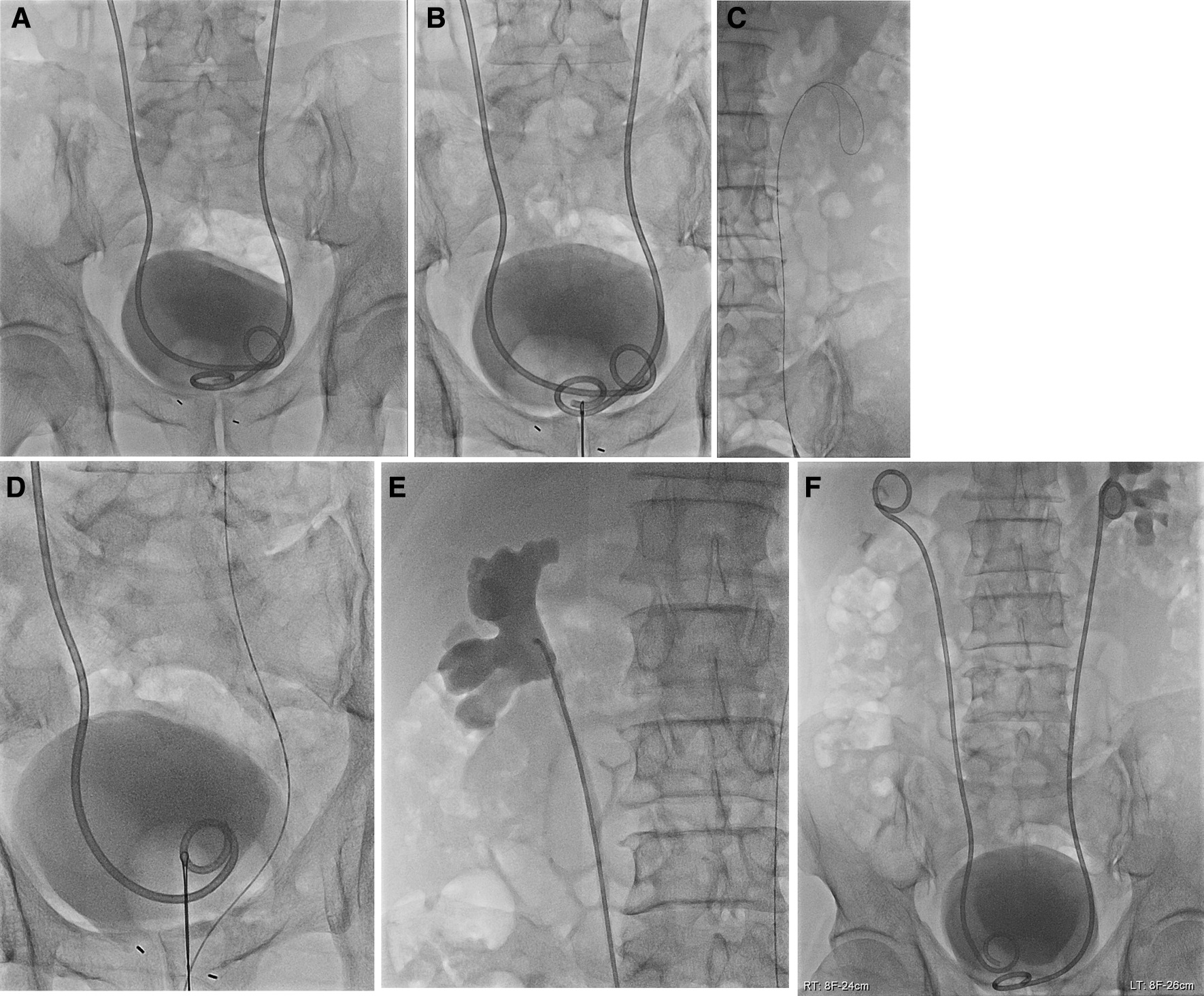
The bladder is filled with diluted intravenous contrast material (**A**). The distal J of the double-J ureteric stent is grabbed (**B**). The distal ureteric stent is cannulated with a hydrophilic 0.035-inch guidewire, which was introduced through the stent to the renal pelvis (**C**). The distal J of the opposite ureteric stent is grabbed (**D**). The ureteric stent is removed, and a 5F Kumpe catheter is advanced over the hydrophilic wire into the renal pelvis. A pyelogram was obtained after injecting contrast material through the catheter (**E**). An 8F DJ ureteric stent is advanced into the collecting system through the stiff wire (**F**)

An 8F DJ ureteric stent (Flexima Ureteral Stent; Boston Scientific Corp, Natick, MA, USA) was advanced into the urinary collecting system through the stiff guidewire. The pigtails were deployed, and the indwelling wires were removed. According to the length between renal pelvis and urinary bladder observed on pyelogram, different stent lengths such as 22 cm, 24 cm, and 26 cm were chosen (Fig. 2).

**Fig. 3:**
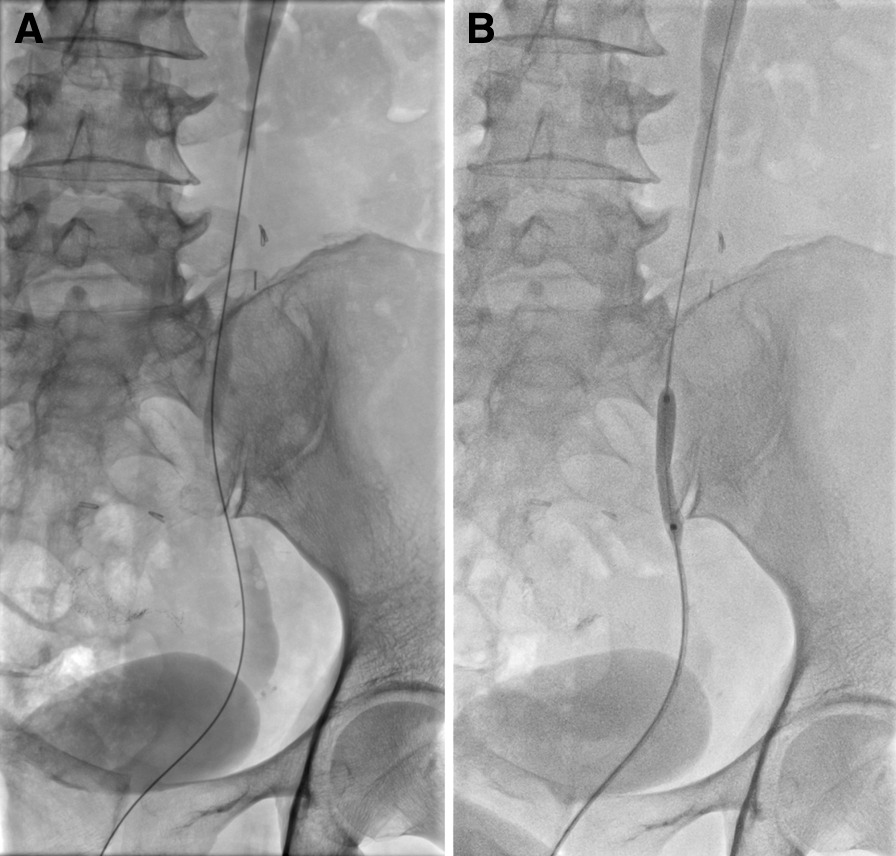
Pre-dilation using a balloon catheter was performed (**B**) in patients with severe ureteral stenosis (**A**)

If a severe ureteral stenosis was observed on pyelogram, DJ stent was introduced after pre-dilation using a urinary balloon catheter (UroMax Ultra; Boston, MA, USA) (Fig. 3).

### Statistical analysis

The method of generalized estimating equations was used to analyze the data, and all statistical analyses were performed using SPSS version 20.0 (Chicago, IL, USA). A *p* value < 0.05 was considered statistically significant.

### Outcomes

Patient outcome was defined as the technical and clinical success of the procedure. Technical success was defined as the successful replacement of ureteral stent in the interventional suite. Clinical success was defined as the satisfactory drainage of the renal pelvis for at least 30 days [3].

Major and minor complications were analyzed according to the quality improvement guidelines for percutaneous nephrostomy (SIR) [4]. Procedure time was defined as the time from obtaining the initial scout image to the end of the procedure, i.e., the deployment of the distal loop of the DJ stent.

## Results

A total of 143 stents were exchanged. In 14 patients, 78 bilateral DJ stents were exchanged in 39 sessions. The remaining 65 unilateral DJ stents were exchanged in 65 sessions. Fifteen patients underwent single-session procedures, whereas 26 patients underwent two or more sessions of the procedures. Thirty-four outpatient-based procedures were performed in 25 sessions, whereas 99 inpatient-based procedures were conducted in 79 sessions. Mean procedure time was 21.6 min (range 9–192 min), and mean procedure interval was 101.8 days (range 5–306 days). Technical success was achieved in 94.4% (135/143) procedures. Technical failures occurred due to the formation of massive urine encrustation in four cases, small bladder capacity in two cases, significant ureteral stenosis in one case, and guide wire loss during DJ stent withdrawal in one case. In three cases, pre-dilation using a balloon catheter was performed before DJ stent insertion. Clinical success was achieved in 93.3% (126/135) procedures. In the nine clinically failed procedures, percutaneous nephrostomy was performed, and the DJ stent was removed via a trans-urethral approach.

Analysis using generalized estimating equation showed that male sex and obstruction at the pelvic ureter negatively affected technical success (Table 2).

**Table 2:** Analysis of GEE parameter estimates to identify the factors affecting the technical success of fluoroscopy-guided trans-urethral exchange of double-J ureteral stents

Parameters		Estimate	Standard error	95% confidence limits		Z	Pr >|Z|	95% confidence limits		
								OR	LL	UL
Intercept		− 0.6838	0.8586	− 2.3667	0.999	− 0.8	0.4258	0.505	0.094	2.716
Sex	Female	3.4145	1.1109	1.2371	5.5919	3.07	0.0021	30.402	3.446	268.235
Sex	Male	0	0	0	0					
Change		0.8713	0.4174	0.0532	1.6894	2.09	0.0369	2.390	1.055	5.416
Obstruction level	Abdominal, intravesical	2.2188	0.8308	0.5904	3.8471	2.67	0.0076	9.196	1.805	46.860
Obstruction level	Pelvic	0	0	0	0					

On the other hand, previous successful exchange positively influenced technical success. Left-sided ureteral stent placement and old age negatively affected clinical success (Table 3).

**Table 3:** Analysis of GEE parameter estimates to identify the factors affecting the clinical success of fluoroscopy-guided trans-urethral exchange of double-J ureteral stents

Parameters		Estimate	Standard error	95% confidence limits		Z	Pr >|Z|	95% confidence limits		
								OR	LL	UL
Intercept		6.3622	1.8523	2.7318	9.9926	3.43	0.0006	579.5199	15.3589	21,866.4330
Sex	Male	1.5405	1.2785	− 0.9654	4.0463	1.2	0.2283	4.6669	0.3808	57.1889
Sex	Female	0	0	0	0					
Side	Right	0.9425	0.418	0.1232	1.7618	2.25	0.0241	2.5664	1.1311	5.8228
Side	Left	0	0	0	0					
Age		− 0.0689	0.0249	− 0.1178	− 0.0201	− 2.76	0.0057	0.9334	0.8890	0.9801

Septic shock occurred in one patient. A 71-year-old male patient with prostate cancer complained of flank pain resulting from DJ stent encrustation during retrieval. The encrusted DJ stent was gently pulled out, and a new DJ stent was inserted successfully. Two hours after the procedure, the patient complained of fever and chills; 12 h later, his blood pressure dropped. Therefore, he was diagnosed with sepsis caused by urinary tract infection. His blood pressure returned to normal after the administration intravenous normal saline and antibiotics. No patient developed hemorrhagic complication requiring transfusion.

## Discussion

The actual overall incidence of ureteral obstruction due to malignancies is unknown; however, it is frequently encountered as a progressive clinical pathology.

Cystoscopy-guided retrograde ureteral stent insertion is a challenging procedure, even for the most experienced urologists, with a mean failure rate of 15.0–27.5% [5]. The cystoscopic approach is unfeasible or fails in patients with “frozen pelvis” or bladder-neck sclerosis, malignant obstructions involving the ureteral orifice, ankylosis who are unable to assume the lithotomy position for cystoscope insertion, bleeding-prone bladder neoplasms, and a urostomy [6].

Another advantage of fluoroscopy-guided trans-urethral replacement of DJ stents is the pronounced relief of pain and discomfort that lead to the avoidance of general anesthesia during the procedure. According to some studies, lidocaine jelly lubrication and lidocaine injection no longer provide effective pain relief during cystoscopy [7, 8]. The use of a smaller, softer silicone Nelaton catheter with lidocaine jelly helps to control pain and avoid general anesthesia during the procedure.

**Fig. 4:**
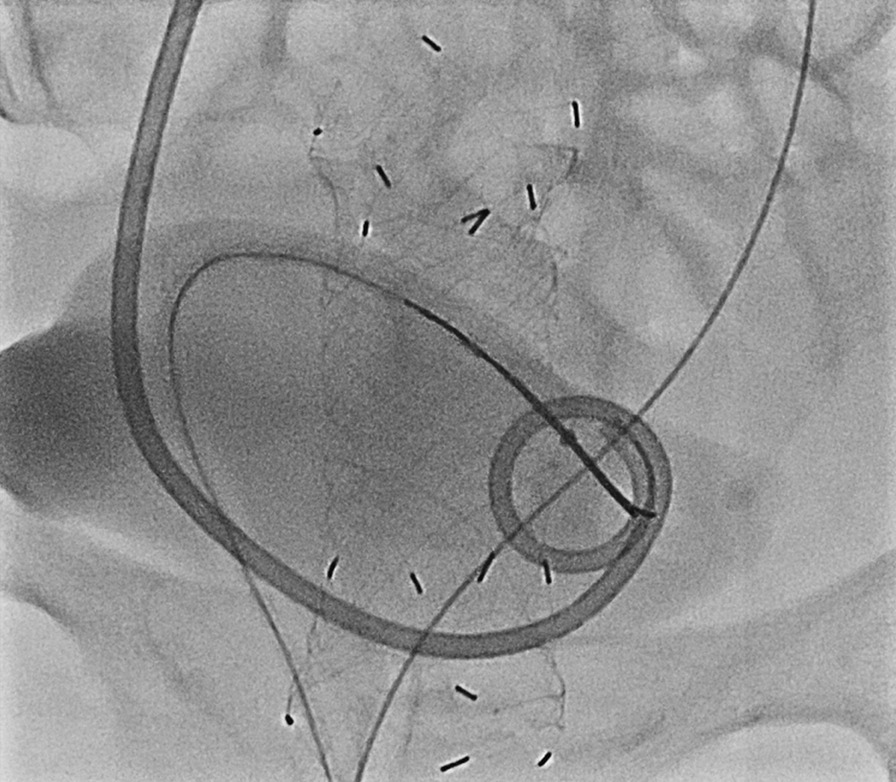
The flexibility of the Nelaton catheter is useful for minimizing patient discomfort during anterior, caudal reorientation of the snare catheter

In previous studies, various devices, such as 14F and 12F Foley catheter, 7F vascular sheath, 7F Nelaton catheter, and 9F Teflon hockey-stick catheter, were used to cannulate the urethra [1, 2, 6, 9]. In this study, an 8F Nelaton catheter was used to cannulate the urethra and as a sheath in both male and female patients. Since Nelaton catheter is a soft, flexible, round-ended catheter, it is considered to cause less discomfort and pain in the curved male urethra. In addition, as a sheath and supporting catheter, Nelaton catheter may provide assistance during the reorientation of the snare catheter in the bladder causing less discomfort and pain (Fig. 4).

In contrast, Nelaton catheter is radiolucent; therefore, careful manipulation is needed during the procedure.

In this study, male sex reduced technical success. The long, curved urethra and the possibility of urethral narrowing due to prostatic hypertrophy may affect the technical success rate in male patients. Among 8 cases of technical failure, there were 6 cases of pelvic ureter obstruction and 2 cases of intravesical obstruction. Of the six cases of technical failure in pelvic ureter obstruction, four were due to urinary encrustation and debris. We removed the stent and performed a new percutaneous nephrostomy in the above cases and counted it as a technical failure. Similar to the results of our study, Wu et al. [10] reported that lower ureter structure has a significant effect on ureter stent failure. Meanwhile, Izumi et al. [11] reported that the obstruction level of the ureter did not affect the stent failure. This result appears to be due to the small sample of the patient population and the fact that most of the obstruction levels were pelvic ureters.

However, further in-depth research is needed to identify the factors affecting fluoroscopy-guided trans-urethral exchange of DJ ureteral stents.

Among 9 cases of clinical failure, there were 6 cases of left ureteral stent and 3 cases of right ureteral stent. Of the 6 clinical failure cases, 4 occurred 2 consecutive times in 2 patients. It was due to uncontrolled urinary tract infection. This seems to have affected the low clinical success of the left ureter stent.

This study has some limitations, mostly originating from its small sample size and retrospective design.

In conclusion, fluoroscopy-guided transurethral exchange of DJ ureteral stents is an effective and less painful procedure than cystoscopic exchange of DJ ureteral stents. As a sheath and supporting catheter, Nelaton catheter is useful to reduce pain and discomfort during the procedure.

## Data Availability

All data generated or analysed during this study are included in this published article.

## References

[1] Carrafiello G, Coppola A, De Marchi G, Fontana F, Piacentino F, Petrillo M, Taborelli A, Angileri SA, Xhepa G, Macchione N (2018). Trans-urethral ureteral stent replacement technique (TRUST): 10-year experience in 1168 patients. Cardiovasc Intervent Radiol.

[2] de Baere T, Denys A, Pappas P, Challier E, Roche A (1994). Ureteral stents: exchange under fluoroscopic control as an effective alternative to cystoscopy. Radiology.

[3] McCarthy E, Kavanagh J, McKernan S, O'Mahony N, McEniff N, Ryan JM, Guiney M (2015). Fluoroscopically guided transurethral removal and/or replacement of ureteric stents in women. Acta Radiol.

[4] Pabon-Ramos WM, Dariushnia SR, Walker TG, d'Othee BJ, Ganguli S, Midia M, Siddiqi N, Kalva SP, Nikolic B (2016). Society of interventional radiology standards of practice C: quality improvement guidelines for percutaneous nephrostomy. J Vasc Interv Radiol.

[5] Wang JY, Zhang HL, Zhu Y, Qin XJ, Dai BO, Ye DW (2016). Predicting the failure of retrograde ureteral stent insertion for managing malignant ureteral obstruction in outpatients. Oncol Lett.

[6] Carrafiello G, Lagana D, Mangini M, Recaldini C, Dizonno M, Giorgianni A, Lumia D, Taborelli A, Cuffari S, Fugazzola C (2007). Fluoroscopically guided retrograde replacement of ureteral stents. Radiol Med.

[7] Kobayashi T, Nishizawa K, Mitsumori K, Ogura K (2004). Instillation of anesthetic gel is no longer necessary in the era of flexible cystoscopy: a crossover study. J Endourol.

[8] McFarlane N, Denstedt J, Ganapathy S, Razvi H (2001). Randomized trial of 10 mL and 20 mL of 2% intraurethral lidocaine gel and placebo in men undergoing flexible cystoscopy. J Endourol.

[9] Park SW, Cha IH, Hong SJ, Yi JG, Jeon HJ, Park JH, Park SJ (2007). Fluoroscopy-guided transurethral removal and exchange of ureteral stents in female patients: technical notes. J Vasc Interv Radiol.

[10] Wu KJ, Chen YZ, Chen M, Chen YH (2021). Clinical factors predicting ureteral stent failure in patients with external ureteral compression. Open Med (Wars).

[11] Izumi K, Mizokami A, Maeda Y, Koh E, Namiki M (2011). Current outcome of patients with ureteral stents for the management of malignant ureteral obstruction. J Urol.

